# High-Throughput Screen for Inhibitors of the Type IV Pilus Assembly ATPase PilB

**DOI:** 10.1128/mSphere.00129-21

**Published:** 2021-03-03

**Authors:** Keane J. Dye, Nancy J. Vogelaar, Pablo Sobrado, Zhaomin Yang

**Affiliations:** a Department of Biological Sciences, Virginia Tech, Blacksburg, Virginia, USA; b Virginia Tech Center for Drug Discovery, Virginia Tech, Blacksburg, Virginia, USA; c Department of Biochemistry, Virginia Tech, Blacksburg, Virginia, USA; University of Rochester

**Keywords:** PilB ATPase, type IV pili (T4P), high-throughput screen (HTS), antivirulence, quercetin, motility, *Myxococcus xanthus*

## Abstract

The bacterial type IV pilus (T4P) is a prominent virulence factor in many significant human pathogens, some of which have become increasingly antibiotic resistant. Antivirulence chemotherapeutics are considered a promising alternative to antibiotics because they target the disease process instead of bacterial viability. However, a roadblock to the discovery of anti-T4P compounds is the lack of a high-throughput screen (HTS) that can be implemented relatively easily and economically. Here, we describe the first HTS for the identification of inhibitors specifically against the T4P assembly ATPase PilB *in vitro*. Chloracidobacterium thermophilum PilB (*Ct*PilB) had been demonstrated to have robust ATPase activity and the ability to bind its expected ligands *in vitro.* We utilized *Ct*PilB and MANT-ATP, a fluorescent ATP analog, to develop a binding assay and adapted it for an HTS. As a proof of principle, we performed a pilot screen with a small compound library of kinase inhibitors and identified quercetin as a PilB inhibitor *in vitro*. Using Myxococcus xanthus as a model bacterium, we found quercetin to reduce its T4P-dependent motility and T4P assembly *in vivo.* These results validated our HTS as effective in identifying PilB inhibitors. This assay may prove valuable in seeking leads for the development of antivirulence chemotherapeutics against PilB, an essential and universal component of all bacterial T4P systems.

**IMPORTANCE** Many bacterial pathogens use their type IV pili (T4P) to facilitate and maintain infection of a human host. Small chemical compounds that inhibit the production or assembly of T4P hold promise in the treatment and prevention of infections, especially in the era of increasing threats from antibiotic-resistant bacteria. However, few chemicals are known to have inhibitory or anti-T4P activity. Their identification has not been easy due to the lack of a method for the screening of compound collections or libraries on a large scale. Here, we report the development of an assay that can be scaled up to screen compound libraries for inhibitors of a critical T4P assembly protein. We further demonstrate that it is feasible to use whole cells to examine potential inhibitors for their activity against T4P assembly in a bacterium.

## INTRODUCTION

The bacterial type IV pilus (T4P), a protein polymer comprised of thousands of pilins (1), is a virulence factor in many pathogens (2–4) and a target for potential chemotherapeutics for disease intervention (5). The T4P filament can extend several micrometers from the cell body and is primarily used as an adhesin during the infection cycle by a bacterial pathogen (2–4, 6). As a virulence factor, the T4P facilitates the adherence of a bacterium to the surface of host cells or a medical device to initiate infections (6–8). In some cases, this leads to the development of biofilms, which can transition an acute infection to a chronic one (9–11). There is also evidence that the physical contact of T4P with host cells or surfaces can regulate the expression of other virulence factors (9, 11–14). The loss of T4P has been shown to significantly attenuate or compromise the virulence process of pathogenic bacteria such as Acinetobacter baumannii, Clostridioides difficile, pathogenic Escherichia coli, Francisella tularensis, Neisseria gonorrhoeae, Pseudomonas aeruginosa, and Vibrio cholerae (3, 4, 15–18). Because the T4P is integral to the infection process for a diverse range of bacterial pathogens, it provides a potential target for the development of therapies against their infections.

The assembly of pilins into the T4P filament requires the T4P machinery (T4PM), which is comprised of a dozen highly conserved T4P or Pil proteins (19). Among them is the cytoplasmic PilB ATPase, commonly known as the T4P assembly or extension motor (20, 21). It hydrolyzes ATP to power the incorporation of pilins into a growing pilus at the base of the T4PM. PilT, another ATPase present in many (but not all) T4P systems, is known as the T4P disassembly or retraction motor (20). It uses ATP as the energy source to disassemble T4P by retraction. The recurrent cycles of T4P extension and retraction catalyzed by PilB and PilT can result in motility in bacteria such as *Neisseria*, P. aeruginosa, and Myxococcus xanthus (22–24). This form of motility is known as bacterial twitching or M. xanthus social (S) gliding (23, 25). A lack of PilB results in the absence of T4P, while elimination of PilT leads to hyperpiliation because of defects in T4P retraction (20, 26). As a result, *pilB* as well as *pilT* mutants have no T4P-dependent motility, providing a convenient assay for the functionality of the T4PM in bacteria with this form of motility (20, 26).

Antivirulence compounds are small molecules that inhibit or interfere with the function or expression of virulence factors such as the T4P (27, 28). As far as we are aware, there have been only two reports of anti-T4P compounds in the literature, both of which appeared in 2019 (29, 30). In one case (29), the antipsychotic drug trifluoperazine and other related phenothiazines were serendipitously discovered because they induced the dispersion of Neisseria meningitidis microaggregates by decreasing T4P levels. Using primary endothelial cells and brain vessels of human origin as well as a humanized mouse model, these compounds were found to reduce bacterial colonization as well as bacterially induced cell injury and vascular lesions (29). In a mouse infection model, phenothiazines were found to provide adjunctive benefits when administered alongside antibiotics (29). Genetic studies identified the target of these anti-T4P compounds as the Na^+^ pumping NADH-ubiquinone oxidoreductase (Na^+^-NQR) complex, which was not known to be involved in T4P dynamics prior to this report (29). In the other case (30), P4MP4 {1-[(piperidin-4-yl)methyl]piperidin-4-ol} (5) was identified from a library of 2,239 compounds by a high-throughput screen (HTS) based on the reduction of N. meningitidis adhesion to cultured cells. Coincidentally, P4MP4 affected N. meningitidis aggregation and T4P in a manner resembling that of phenothiazines despite their different routes of discovery. Biochemical studies in this investigation pointed to PilF, the PilB equivalent in the N. meningitidis T4P system, as the potential target of P4MP4 instead of the Na^+^-NQR complex (30). It should be emphasized that the T4PM was not specifically targeted by the antiaggregation (29) or the antiadhesion (30) assays in either case for the discovery of phenothiazines or P4MP4. The convergence of their effects on the T4P strongly substantiated the T4PM as a valuable target for the development of antivirulence chemotherapeutics (5).

We report here the development of an HTS for the identification of compounds that inhibit the PilB ATPase specifically. Our previous work with Chloracidobacterium thermophilum PilB (*Ct*PilB) showed that it is a robust ATPase (31) that binds the secondary messenger c-di-GMP with a critical role in biofilm regulation (32). Here, we demonstrate that the fluorescent ATP analog MANT-ATP also binds *Ct*PilB and that its fluorescence is significantly increased by this association. We developed an assay for the identification of compounds that reduced MANT-ATP binding to *Ct*PilB, thereby causing a decrease in MANT-ATP fluorescence. After optimization and adaptation of this assay for HTS, we identified quercetin from a small-compound library of kinase inhibitors. Biochemical assays confirmed that quercetin inhibits both the binding of *Ct*PilB with MANT-ATP and its ATPase activity *in vitro*. Experiments using M. xanthus as a model further indicated that quercetin reduced the expansion of M. xanthus colonies by its T4P-dependent S motility. Additional results suggested that the inhibitory effect of quercetin on M. xanthus S motility is related to diminished T4P assembly, supporting the conclusion that quercetin functions as an inhibitor of the PilB assembly ATPase *in vivo*. These results collectively illustrate that our newly developed HTS is able to identify compounds *in vitro* that can be effective *in vivo* to facilitate the development of antivirulence chemotherapeutics against bacterial pathogens with T4P as a virulence factor.

## RESULTS

### ATP and its fluorescent analog MANT-ATP compete for binding to PilB.

**FIG 1:**
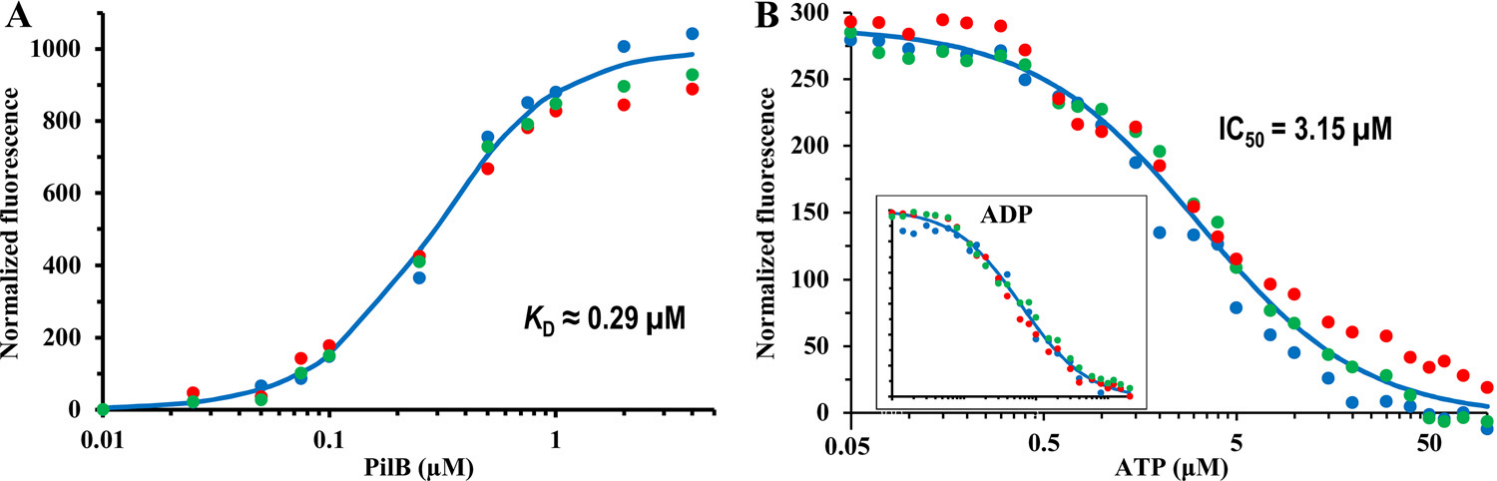
MANT-ATP and ATP compete for binding to *Ct*PilB. For both panels, the averages from three independent experiments are shown as three sets of different colored dots, with the standard deviation omitted for clarity. (A) Binding between MANT-ATP and *Ct*PilB. Different concentrations of *Ct*PilB were incubated with 0.20 μM MANT-ATP in triplicates, and the fluorescence was quantified. The fit of these data to an isotherm as described in M&M resulted in a dissociation constant (*K_D_*) of 0.29 ± 0.01 μM. (B) Competition between ATP and MANT-ATP for *Ct*PilB binding. *Ct*PilB at 0.25 μM and MANT-ATP at 0.125 μM were incubated with various concentrations of ATP (or ADP [inset]) in triplicates, and the fluorescence of the samples was quantified. Isotherms with best fit to the data sets resulted in an IC_50_ of 3.15 μM for ATP and 3.07 μM for ADP.

*Ct*PilB was used previously to analyze its binding of ATP, ADP, and ATP-γ-S by isothermal calorimetry (ITC) (32). An alternative for such analysis is to use fluorescent nucleotide analogs, such as MANT-ATP, which may emit enhanced fluorescence when bound to proteins (33, 34). We examined the binding between *Ct*PilB and MANT-ATP as described in Materials and Methods (M&M). As shown in Fig. 1A, increasing concentrations of *Ct*PilB led to increased fluorescence of MANT-ATP, which was kept at a constant concentration. A binding isotherm fitted to this data set produced a dissociation constant (*K_D_*) of 0.29 ± 0.01 μM for the binding of these two partners. These results indicate that the fluorescence of MANT-ATP can be used to analyze its binding with *Ct*PilB.

The above *K_D_* value of 0.29 μM for MANT-ATP and *Ct*PilB binding is approximately 10-fold lower than the *K*_D_ of 3.8 μM for that between ATP and *Ct*PilB determined by ITC (32). This increase in affinity for an ATP analog was similarly observed between protein kinases and TNP-ATP (35, 36), possibly due to additional interactions of the fluorophore of a fluorescent ATP analog with the protein (37). If MANT-ATP occupies the orthosteric pocket as ATP as expected, it should behave as a competitive ligand or inhibitor. If so, the *K_D_* for ATP could be determined by its inhibition constant (*K_i_*) on MANT-ATP and *Ct*PilB binding. As shown in Fig. 1B, when ATP was included in reaction mixtures containing both MANT-ATP and *Ct*PilB, the fluorescent signal decreased with increasing concentrations of ATP. The half maximal inhibitory concentration (IC_50_) of ATP on MANT-ATP binding was determined to be 3.15 μM. The resulting *K_i_* is 2.20 μM, close to the previously published *K_D_* value (32). Likewise, ADP also competed with MANT-ATP for *Ct*PilB binding as indicated by the decline in the fluorescence signal with increasing ADP concentrations (inset in Fig. 1B). The ability of both ATP and ADP to compete with MANT-ATP agrees with previously published observations, which showed that both nucleotides bind to *Ct*PilB with similar affinities (32). AMP up to 100 μM failed to compete with MANT-ATP for *Ct*PilB binding (data not shown). This is consistent with observations that both the γ- and β-phosphates of ATP are crucial for interactions with PilB (21, 38, 39). In summary, these results show that MANT-ATP can be used as a fluorescent probe to analyze the binding of *Ct*PilB to its cognate nucleotide ligands.

### Development and implementation of an HTS identified quercetin as a PilB inhibitor.

**FIG 2:**
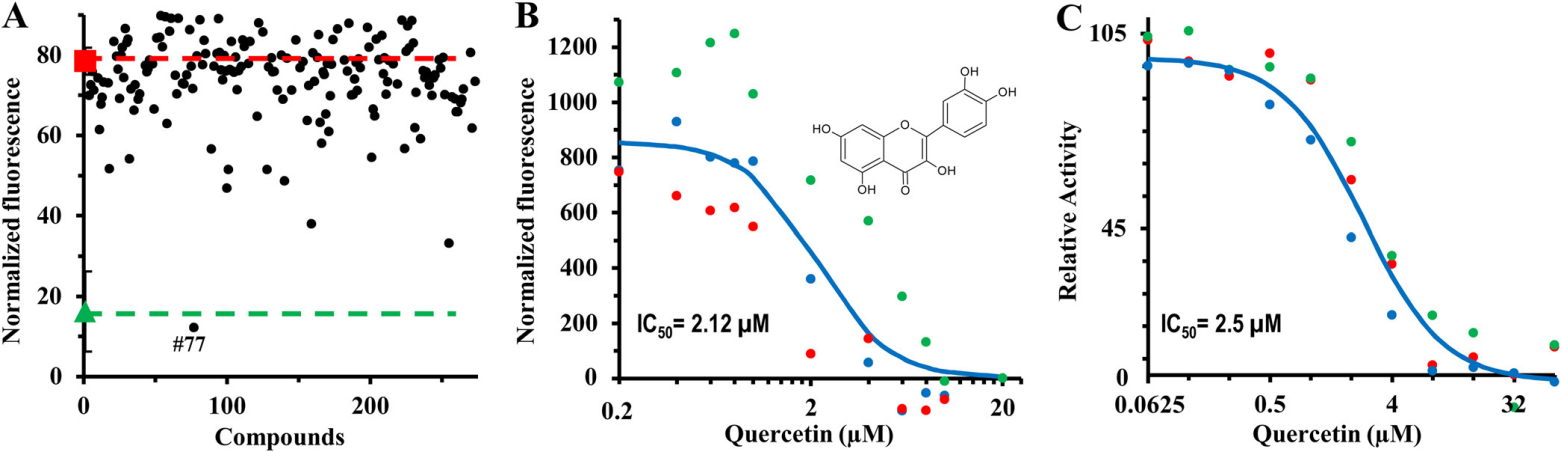
Quercetin inhibits *Ct*PilB. (A) An HTS identified quercetin as an inhibitor of MANT-ATP binding to *Ct*PilB. Library compounds were screened by a high-throughput screen as described in the main text for chemicals that reduced the ability of PilB to associate with MANT-ATP. The red and green lines indicate the levels of fluorescence for the negative and positive controls, respectively. The former included MANT-ATP and *Ct*PilB, while the latter included 20 μM ATP in addition. Compound 77, which is quercetin, was found to reduce the fluorescence to a level similar to that of ATP. The maximum value for the *y* axis here is 90, and compounds that emit significant fluorescence are cut off from this figure. (B) Quercetin reduces MANT-ATP binding to *Ct*PilB. The fluorescence of triplicate samples containing constant concentrations of *Ct*PilB and MANT-ATP with various concentrations of quercetin was analyzed. The three different sets of colored dots represent the averages from three independent experiments, with error bars omitted for clarity. Shown is the best fit of the data to an isotherm with an IC_50_ of 2.12 μM (see M&M). The structure of quercetin is shown in the inset. (C) Quercetin inhibits the ATPase activity of *Ct*PilB. The ATPase activity of *Ct*PilB was determined in triplicates in the presence of quercetin at the indicated concentrations by endpoint assays. The three different sets of colored dots show the averages from three independent experiments, with errors bars omitted for clarity. The IC_50_ of quercetin against *Ct*PilB ATPase activity was determined to be 2.50 μM by curve fitting.

We reasoned that if a compound could reduce the binding of ATP to PilB, it would inhibit the ATPase activity of PilB. Such an inhibitor could be identified from a compound library by HTS based on its ability to reduce the binding of MANT-ATP to *Ct*PilB. ATP, which clearly competes with MANT-ATP for *Ct*PilB binding (Fig. 1B), was used as a positive control to develop an HTS in a 384-well microtiter plate format. The HTS included 0.4 μM MANT-ATP and 0.5 μM *Ct*PilB in a total volume of 20 μl per well. Using this assay, we screened a Selleckchem compound library of 273 kinase inhibitors. For the library compounds, a final concentration of 20 μM was used for the HTS. Included in the screen were four sets of controls, each in multiple wells at different positions on the screen plate and all with the same concentration of dimethyl sulfoxide (DMSO) as the compound wells. The first set, the positive control, contained *Ct*PilB and MANT-ATP with ATP as the inhibitory or competitive ligand. The second set, the negative controls, contained *Ct*PilB and MANT-ATP without ATP. The last two, which were used for normalization, contained either *Ct*PilB or MANT-ATP alone. An acceptable Z′ factor (40) of 0.57 was calculated for this HTS. As shown in Fig. 2A, although a few library compounds lowered the fluorescence relative to the negative control, compound 77 stood out in that it decreased the fluorescence to levels similar to those of the positive control with ATP. This compound is quercetin, a flavonoid of plant origin that inhibits the activity of protein kinases (41) as well as ATPases (42, 43). These results suggest that our HTS is effective for the identification of PilB inhibitors and that quercetin could inhibit *Ct*PilB as it does other ATPases (42, 43).

### Quercetin inhibits the ATPase activity of *Ct*PilB *in vitro*.

We examined by biochemical experiments if quercetin could indeed inhibit the binding of *Ct*PilB to MANTP-ATP as described earlier (see Fig. 1B). As shown in Fig. 2B, the fluorescence of MANT-ATP in the presence of *Ct*PilB was clearly diminished by quercetin in a dosage-dependent manner. It reduced the fluorescence to the baseline when quercetin was at or above 10 μM. The IC_50_ of quercetin was determined to be 2.12 μM, showing that quercetin inhibits the binding of PilB to MANT-ATP with a potency similar to that of ATP, which had an IC_50_ of 3.15 μM in this assay (Fig. 1B).

To investigate if quercetin could inhibit the ATPase activity of *Ct*PilB, we analyzed the ATPase activity of *Ct*PilB (31) in the presence of quercetin. As shown in Fig. 2C, the activity of *Ct*PilB declined in the presence of quercetin in a dosage-dependent manner. ATP hydrolysis by *Ct*PilB was completely inhibited by quercetin at or above 32 μM. The IC_50_ for this inhibition is estimated to be about 2.50 μM, which is similar to 2.12 μM, the IC_50_ for the inhibition of MANT-ATP binding to *Ct*PilB (Fig. 2B). We conclude that quercetin is a potent inhibitor of *Ct*PilB ATPase *in vitro*, demonstrating that our newly developed HTS is effective in identifying PilB inhibitors from a compound library (Fig. 2A).

### Quercetin impedes T4P-mediated bacterial motility *in vivo*.

**FIG 3:**
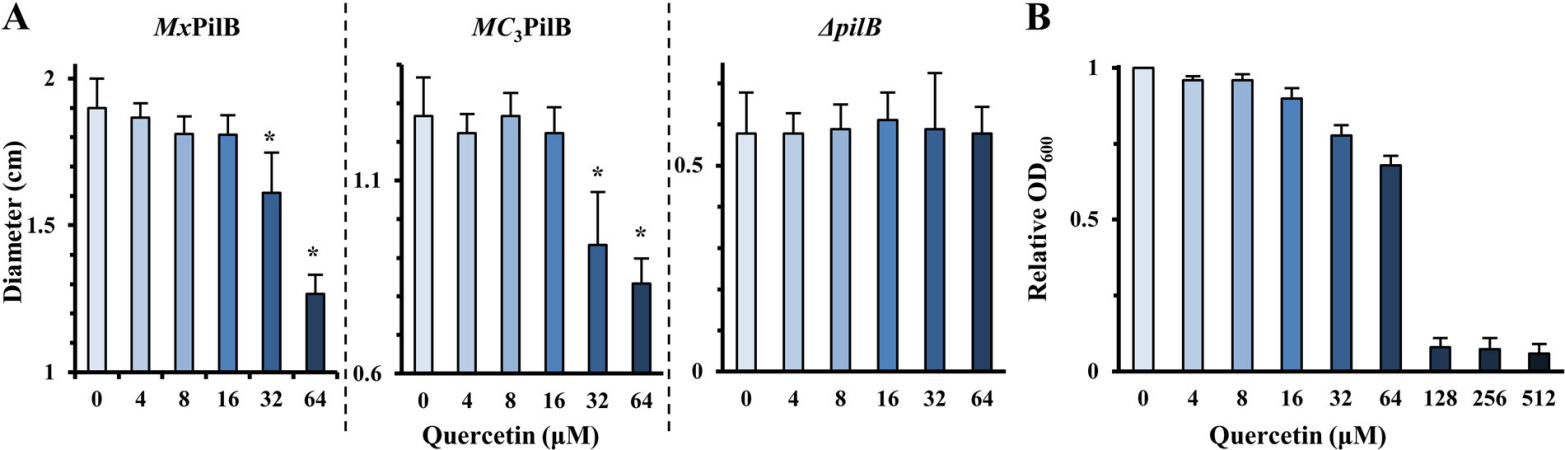
Quercetin inhibits T4P-mediated motility in M. xanthus. (A) Quercetin reduces T4P-mediated motility. Shown are the averages of colony diameters of *Mx*PilB (YZ1674), *MC*_3_PilB (YZ2232), and Δ*pilB* (DK10416) strains on soft agar plates, with standard deviations as error bars. The average for a given strain was calculated from the measurement of 12 colonies at a given concentration of quercetin. Asterisks signify that the values at the indicated concentration of quercetin are statistically different from that without quercetin, with *P* values of <0.05 from Student’s *t* test. (B) Growth inhibition of M. xanthus in liquid culture by quercetin. Liquid cultures in triplicates were grown in the presence of quercetin at the indicated concentrations, and OD_600_ was normalized to the culture without quercetin. Shown here are the averages and standard deviations (error bars) from three independent experiments. The MIC of quercetin against M. xanthus is about 128 μM.

We next examined if quercetin has the ability to impact functions associated with T4P *in vivo* using M. xanthus as a model organism. This bacterium has a form of surface motility that is powered by T4P extension and retraction (23, 44). Its T4P-depedent motility, commonly known as S motility, can be conveniently analyzed on plates with low-percentage agar (soft agar) (45). Experiments were conducted with two strains, YZ1674 and YZ2232, both of which produce T4P (T4P^+^) and exhibit the T4P-dependent S motility (31). YZ1674 expresses the endogenous or wild-type (WT) M. xanthus PilB (*Mx*PilB), while YZ2232 expresses *MC*_3_PilB, a PilB chimera with the N terminus of *Mx*PilB and the C-terminal ATPase catalytic domains of *Ct*PilB. The *pilB* deletion (Δ*pilB*) strain DK10416 was used as the negative control, as it is devoid of T4P (T4P^−^) and without S motility. These M. xanthus strains were tested for their T4P-mediated motility on soft agar plates with different concentrations of quercetin. As shown in Fig. 3A and Fig. S1 in the supplemental material, quercetin at or above 32 μM significantly reduced the spreading of the *Mx*PilB-expressing (YZ1674) and the *MC*_3_PilB-expressing (YZ2232) strains to comparable extents. The results are consistent with earlier observations that quercetin at the same concentrations inhibited both MANT-ATP binding and the ATPase activity of *Ct*PilB (Fig. 2B and 2C). The results from the motility assays suggest that quercetin is capable of inhibiting the activity of PilB as the T4P assembly ATPase in M. xanthus *in vivo*.

10.1128/mSphere.00129-21.1 FIG S1 Quercetin inhabits M. xanthus colony expansion by T4P-depedent motility. Five microliters of cell suspensions of YZ1674 (*Mx*PilB), YZ2232 (*MC*_3_PilB), and DK10416 (Δ*pilB*) were placed on soft agar plates containing quercetin at the specified concentrations. Photographs of plates were taken after 4 days of incubation at 32°C. Plates with 4 μM and 8 μM quercetin are not shown because their results are comparable to those without quercetin. See text for details of the M. xanthus strains. Download FIG S1, TIF file, 1.3 MB. Copyright © 2021 Dye et al. 2021 Dye et al. https://creativecommons.org/licenses/by/4.0/ This content is distributed under the terms of the Creative Commons Attribution 4.0 International license.

However, quercetin is also known to inhibit the growth of certain bacteria, including Gram-negative bacteria (46). The observed reduction in M. xanthus S motility described above could be due to the inhibition of growth. We examined the effect of quercetin on M. xanthus growth in liquid culture as shown in Fig. 3B. The results indicated that quercetin could inhibit M. xanthus with an estimated MIC of about 128 μM. At the sub-MICs of 32 μM and 64 μM, quercetin also appreciably reduced the growth of M. xanthus in liquid (Fig. 3B). Coincidentally, these are the concentrations at which quercetin affected M. xanthus T4P-dependent or S motility in plate assays as well (Fig. 3A). It is pertinent here to highlight that M. xanthus has long been known to have T4P-dependent and T4P-independent motility systems (23, 47, 48). While the former can be analyzed on soft agar plates, the latter, known as adventurous (A) motility, can be assayed on plates with 1.5% agar (hard agar) (45). We therefore examined the impact of quercetin on the A motility system of M. xanthus on hard agar plates. As shown in Fig. S2 and S3, none of the three M. xanthus strains had their colony expansion impacted by quercetin up to 64 μM. These results argue that the effect of quercetin on M. xanthus T4P-dependent motility is not likely attributable to an effect on growth. Instead, the inhibition of PilB as the T4P assembly ATPase is a more reasonable explanation for the effect of quercetin on M. xanthus S motility.

10.1128/mSphere.00129-21.2 FIG S2 Quercetin has no effect on M. xanthus T4P-indepedent motility. Five microliters of cell suspensions of YZ1674 (*Mx*PilB), YZ2232 (*MC*_3_PilB), and DK10416 (Δ*pilB*) were placed on hard agar plates with quercetin at the specified concentrations. Photographs of plates were taken after 4 days of incubation at 32°C. Plates with 4 μM and 8 μM quercetin are not shown. Download FIG S2, TIF file, 1.4 MB. Copyright © 2021 Dye et al. 2021 Dye et al. https://creativecommons.org/licenses/by/4.0/ This content is distributed under the terms of the Creative Commons Attribution 4.0 International license.

10.1128/mSphere.00129-21.3 FIG S3 Quercetin has no effect on M. xanthus T4P-independent motility. The diameters of 12 colonies on hard agar plates were measured for YZ1674 (*Mx*PilB), YZ2232 (*MC*_3_PilB), and DK10416 (Δ*pilB*) at the given concentrations of quercetin. Shown are the averages and standard deviations (error bars) from these measurements. Download FIG S3, TIF file, 0.04 MB. Copyright © 2021 Dye et al. 2021 Dye et al. https://creativecommons.org/licenses/by/4.0/ This content is distributed under the terms of the Creative Commons Attribution 4.0 International license.

### Quercetin does not impact the EPS level of M. xanthus.

**FIG 4:**
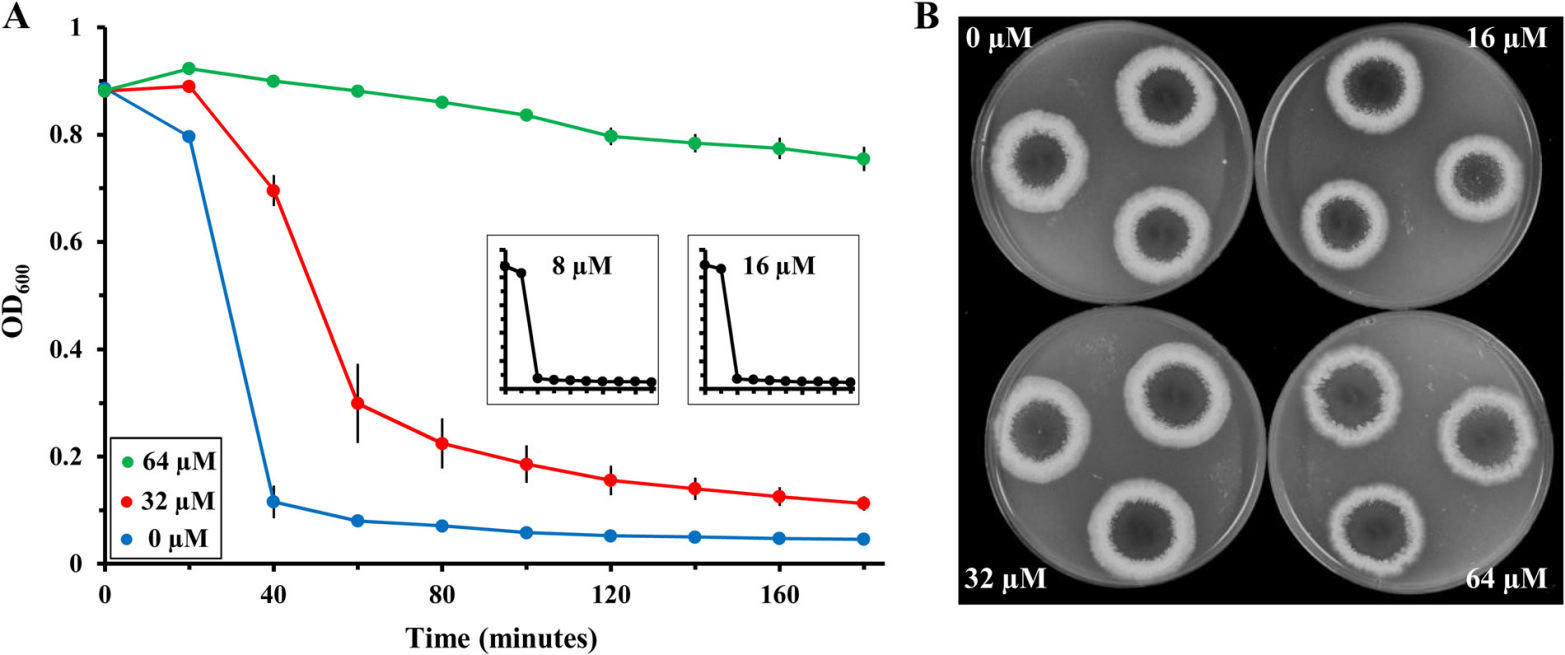
Quercetin blocks agglutination but shows no impact on M. xanthus EPS level. (A) Effect of quercetin on M. xanthus agglutination. OD_600_ of M. xanthus cells in agglutination buffer at an initial OD_600_ of 0.9 were monitored over time in the presence of quercetin at the indicated concentrations. Shown are the averages and standard deviations (error bars) from three independent experiments, each of which was conducted in triplicates. Agglutination with 8 μM and 16 μM quercetin was indistinguishable from the control without quercetin, and their results are shown as insets to avoid curve overlap. (B) Effect of quercetin on M. xanthus EPS levels. EPS levels of M. xanthus in the presence of quercetin at specified concentrations were examined on plates with the fluorescent dye calcofluor white as described in M&M. The intensity of the fluorescence reflects EPS levels in this assay.

It should be noted that the T4P-mediated motility of M. xanthus requires exopolysaccharide (EPS), in addition to the presence of retractable T4P (49). An agglutination assay was performed as a first step to examine the effect of quercetin on EPS because it is required for M. xanthus to agglutinate (49). As shown in Fig. 4A, quercetin up to 16 μM did not affect M. xanthus agglutination. Although M. xanthus agglutinated in the presence of 32 μM quercetin, this occurred at a noticeably lower rate. When quercetin was increased to 64 μM, M. xanthus failed to agglutinate entirely. Because quercetin inhibited agglutination and T4P-mediated motility at similar concentrations (Fig. 3A), its effect on T4P-dependent motility could be through the modulation of EPS, T4P, or both. We next examined M. xanthus EPS levels more directly by a plate assay based on the binding of the fluorescent dye calcofluor white (CW) by M. xanthus EPS (50). Cells were spotted onto CW-containing plates with different concentrations of quercetin, and the fluorescence was examined after incubation for 7 days (51). As show in Fig. 4B, no difference in fluorescence was observed regardless of the concentration of quercetin. These observations suggested that quercetin does not impact EPS levels in M. xanthus on plates and its effect on T4P-depedent motility is consistent with an effect on T4P assembly through the inhibition of PilB. These results are reminiscent of the delayed-agglutination phenotype of an M. xanthus strain with elevated c-di-GMP levels through the expression of an exogenous diguanylate cyclase (52); this strain was found to have reduced levels of piliation with wild-type level of EPS.

### Quercetin inhibits T4P assembly in M. xanthus.

We analyzed the effect of quercetin on T4P assembly more directly using a dot blotting assay under nongrowing conditions (see M&M). In this assay, the pili on M. xanthus were first sheared off mechanically by vortexing cells in a buffer without nutrients (53, 54). These “bald” cells were then allowed to repiliate in the absence or presence of quercetin in the buffer for 20 min. For each treatment, three fractions were prepared and analyzed using anti-pilin antibodies. The T4P fraction contained the sheared pilus as a measure of piliation levels, whereas the “pilin” fraction was the lysate of cells with their pili sheared off. Also included was the whole-cell (WC) lysate from cells with their pili intact as a control. During the protocol development, bald M. xanthus cells were found to restore their piliation near preshearing levels in about 30 min under our experimental conditions (Fig. S4). For later experiments, incubation with quercetin was allowed for 20 min before stoppage of the treatment.

10.1128/mSphere.00129-21.4 FIG S4 Progression of repiliation of bald M. xanthus cells. (A) Bald M. xanthus cells without T4P were incubated in agglutination buffer for repiliation. The T4P fractions were prepared from samples taken at the indicated intervals and analyzed by dot blotting using anti-pilin antibodies. The three rows of samples were from three biological replicates. Each sample was spotted onto the membrane in triplicates in the same row. (B) Quantification of the results in panel A as described in Materials and Methods in the text. The three different sets of colored dots correspond to the three biological replicates. At a given time, there are three data points in the same color representing the triplicate dots on the membrane, with the horizontal bar representing the average. Download FIG S4, TIF file, 0.1 MB. Copyright © 2021 Dye et al. 2021 Dye et al. https://creativecommons.org/licenses/by/4.0/ This content is distributed under the terms of the Creative Commons Attribution 4.0 International license.

**FIG 5:**
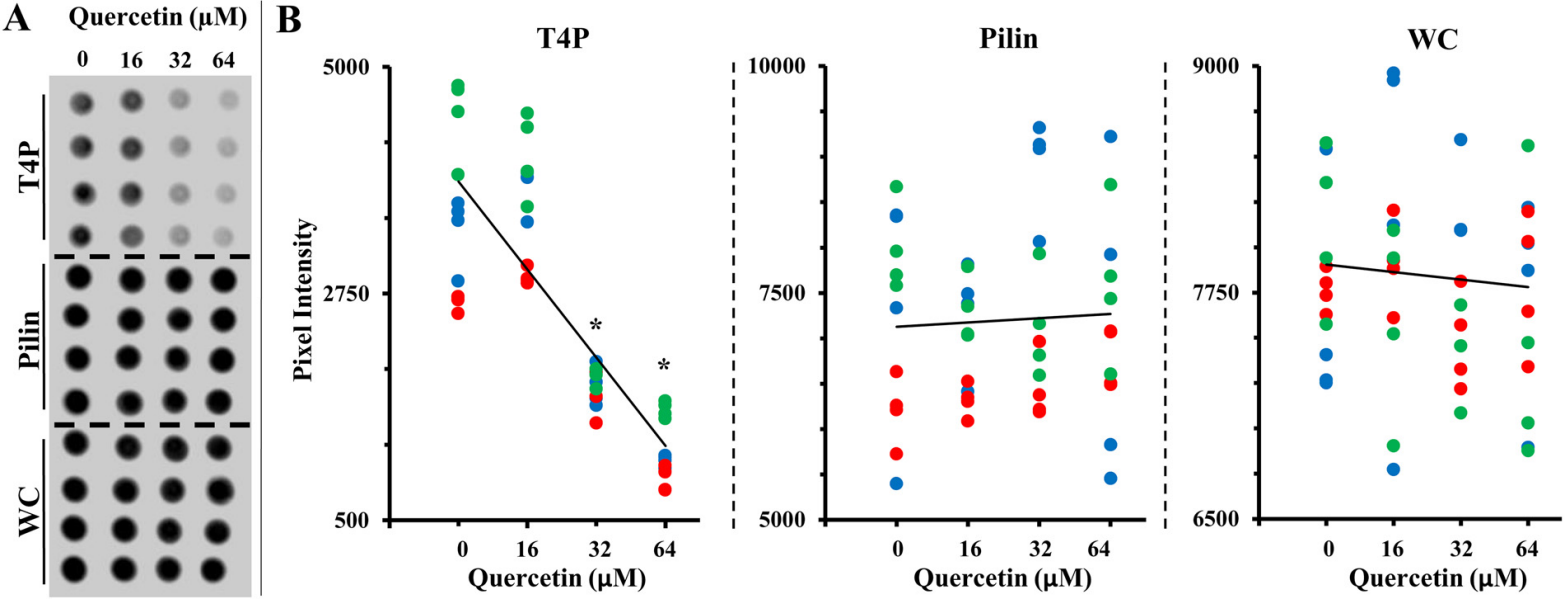
Quercetin inhibits M. xanthus T4P assembly. (A) Dot blot analysis of M. xanthus fractions using anti-pilin antibodies. The T4P fraction contains the assembled T4P that has been sheared off. The pilin fraction is from cells with their T4P sheared off, and the WC (whole-cell) fraction is the lysate of cells with their T4P intact. Four aliquots of the same sample were spotted onto a nitrocellulose membrane in a column and processed for the dot blot assay. Shown here is one representative from multiple experiments with similar results. (B) Quantification of signals from different fractions on dot blots. Shown are the results from three independent experiments, each represented by dots of the same color. At each quercetin concentration, there are four dots of the same color, representing values of quadruplet samples. A linear trend line based on the averages is drawn for visualization purposes. Asterisks indicate that the signals at the specified concentrations of quercetin are statistically different from the control without quercetin, with a *P* value of <0.05.

As shown in Fig. 5A, the signal strength for pilins in the WC and pilin fractions were not affected by the treatment with quercetin, which was confirmed by quantification of the dot blot (Fig. 5B). In contrast, the levels of piliation as indicated by the T4P fraction were clearly reduced by quercetin in a concentration-dependent manner. The signal for T4P in the presence of 16 μM quercetin was not statistically different from that without quercetin. However, the 20-min treatment with 32 μM and 64 μM quercetin significantly reduced piliation levels. At these two higher quercetin concentrations, T4P levels were about 50% and 30% of that without quercetin treatment, respectively (Fig. 5B).

The above-described treatments were conducted in a buffer with no nutrients for bacterial growth in a short time frame. However, because quercetin at 32 μM and 64 μM appreciably decreased the growth of M. xanthus in liquid media over a longer time span (Fig. 3B), it was possible that the quercetin treatment described above, however brief, could impact cell viability or membrane integrity. We used LIVE/DEAD staining to examine the effect of quercetin treatment on cells under the same conditions as for the above-described piliation assay (Fig. 5). As shown in Fig. S5, in the control samples, in which no quercetin was included, 95.7% ± 1.4% cells were found to be viable. The proportions of viable cells treated with 16 μM, 32 μM, and 64 μM quercetin were virtually identical to that of the control at 96.4% ± 0.7%, 96.7% ± 0.8%, and 96.1% ± 0.6%, respectively. These observations show that the effect of quercetin on T4P assembly under our experimental conditions (Fig. 5) is not due to an influence on cell viability or membrane integrity.

10.1128/mSphere.00129-21.5 FIG S5 Quercetin does not affect the viability of M. xanthus cells. The LIVE/DEAD *Bac*Light bacterial viability kit (Invitrogen) was used to assess the effect of quercetin on cell viability or membrane integrity. In LIVE/DEAD staining, cells with their cell membrane intact stain fluorescent green and are scored as live or viable. Those with compromised cell membranes stain fluorescent red and are considered dead or unviable. The same M. xanthus cells were prepared and treated with quercetin as described for the piliation assay (Fig. 5 in the main text). After the 20-min treatment, samples were washed twice with agglutination buffer and stained according to the manufacturer’s protocol. An Olympus IX81 microscope with a Hamamatsu C4742 charge-coupled-device (CCD) camera was used to analyze the samples using the fluorescein isothiocyanate (FITC) and Texas Red filter sets. For imaging, a 10-μl aliquot of a sample was spotted onto an agarose pad between a glass slide and a coverslip. In one experiment, three slides were prepared for each sample, and images were acquired from three positions in a triangular pattern from each slide. ImageJ was used for data analysis. (A) The table shows the average percentage of live cells with standard deviations (SD) from three independent experiments (no. 1, 2, and 3) after treatment with quercetin at the indicated concentrations. The last row show the averages from these three experiments. (B) The averages from the three individual experiments in the table are presented in a bar graph, with the error bar representing SD. The data from the same experiment are represented in the same color. Download FIG S5, TIF file, 0.1 MB. Copyright © 2021 Dye et al. 2021 Dye et al. https://creativecommons.org/licenses/by/4.0/ This content is distributed under the terms of the Creative Commons Attribution 4.0 International license.

Taking into consideration that quercetin was initially identified as an inhibitor of the PilB ATPase *in vitro* (Fig. 2), the observations in this experiment (Fig. S5) and the differential motility assays (Fig. 3) support the conclusion that the inhibition of T4P assembly by quercetin (Fig. 5) likely results from its inhibition of T4P assembly *in vivo* through a direct effect on PilB as the T4P assembly ATPase.

## DISCUSSION

Here, we describe the development of an HTS that allowed the identification of quercetin as an anti-T4P compound targeting the assembly ATPase PilB. This work took advantage of *Ct*PilB, a member of the PilB ATPase family that is amenable to *in vitro* analysis. We utilized its binding to a fluorescent ATP analog to develop an assay adapted for HTS. This led to the discovery and confirmation of quercetin as an inhibitor of *Ct*PilB ATPase activity *in vitro*. Using M. xanthus as a model organism, we demonstrated that quercetin inhibits T4P-dependent motility and T4P assembly *in vivo*. We conclude that our HTS can be effective in the identification of PilB inhibitors from compound libraries for the development of chemotherapies against bacteria with T4P as a virulence factor. It should be noted that in this HTS, about 25% of the library compounds fluoresced at a wavelength similar to that of MANT-ATP (Fig. 2A). Therefore, their ability to inhibit MANT-ATP binding to *Ct*PilB could not be assessed.

With the increasing prevalence of antibiotic resistance in bacterial pathogens, there is a pressing need to explore treatments of bacterial infections other than the use of antibiotics. Antivirulence therapies, which target bacterial virulence factors, are considered a promising alternative. Passive immunotherapies against virulence factors such as the T4P have been explored in animal models (55, 56). Interference with or inhibition of the expression or function of virulence factors by small antivirulence molecules is another approach in the antivirulence strategy (57). Yet chemicals with anti-T4P activities have been few and far between, especially in comparison with those targeting type III secretion systems and bacterial quorum sensing systems (58–61). There had been no knowledge of anti-T4P small molecules before the reports of phenothiazines and P4MP4 in 2019 (29, 30). One of the main challenges was the lack of an HTS for the identification of inhibitors of the T4PM. The discovery of phenothiazines stemmed from the fortuitous observation that the antipsychotic drug trifluoperazine dispersed N. meningitidis cells in microaggregates (29). While P4MP4 was identified in an HTS, the assay focused on the inhibition of N. meningitidis adhesion to cultured cells with extensive imaging requirements (30). These top-down approaches did not target the T4PM specifically and could well have led to targets other than the conserved T4P proteins.

Our assay takes more of a bottom-up approach to specifically target the ubiquitous T4P protein PilB. We used *Ct*PilB as a model enzyme to screen for inhibitors because it is the most active among the canonical PilB ATPases *in vitro* (31). The use of a model protein for inhibitor identification was validated in this study in two M. xanthus strains expressing different PilB variants. One strain (YZ1674) has the wild-type endogenous *Mx*PilB, while the other (YZ2232) has *MC_3_*PilB, a hybrid PilB with the ATPase catalytic core of *Ct*PilB (Fig. 3). It was perhaps not surprising that quercetin inhibited the T4P-dependent motility of YZ2232 (*MC_3_*PilB). More importantly, the T4P-dependent motility of YZ1674 (*Mx*PilB) was similarly inhibited, suggesting that quercetin may interact with and inhibit *Mx*PilB and *Ct*PilB in similar fashions mechanistically. Additional experiments with M. xanthus further substantiated the conclusion that quercetin negatively impacted T4P assembly in this bacterium because quercetin reduced T4P levels on cells with preexisting T4P sheared off. In this context, it is noted that quercetin was reported to inhibit twitching in P. aeruginosa (62). In combination with the observations that phenothiazines and P4MP4 were active in both N. meningitidis and N. gonorrhoeae (5, 30), these results lend credence to the use of *Ct*PilB as a model protein to identify leads for the development of anti-T4P chemotherapeutics against pathogenic bacteria with orthologous PilB proteins.

In summary, we have developed the first known HTS that specifically targets a T4P protein to allow the identification of anti-T4P compounds by a bottom-up approach. This is possible thanks to the availability of *Ct*PilB as a representative of the conserved family of PilB ATPases. The rationale here was that PilB enzymes from most bacteria examined up to date have been recalcitrant to productive biochemical and biophysical analysis *in vitro*, with *Ct*PilB as an exception. It has been demonstrated to be a hexameric ATPase with expected ligand binding capacity and robust ATP hydrolyzing activities (31, 32). The results here and elsewhere support the use of a model protein for the discovery and development of anti-T4P compounds for antivirulence purposes. There is no question that this principle has been applied to the development of antibiotics such as β-lactams against diverse bacteria with phenomenal success.

## MATERIALS AND METHODS

### Strains, growth conditions, chemicals, and miscellaneous methods.

The M. xanthus strains used in this study were DK10416 (Δ*pilB*) (26), YZ603 (Δ*difE*) (50), YZ1674 (*ΔpilB att*::Mx*pilB*) (51), and YZ2232 (Δ*pilB att::*MC_3_*pilB*) (31, 63). Unless stated otherwise, they were maintained and grown on Casitone-yeast extract (CYE) agar plates or media at 32°C. Conditions and procedures for the expression and purification of the *Ct*PilB protein were as previously described (31).

All stock solutions of quercetin used in this study were prepared by dissolving quercetin hydrate (ACROS Organics) in dimethyl sulfoxide (DMSO; Fisher Biotech). Unless otherwise stated, 10× stocks were prepared for each concentration of quercetin in DMSO before their use in experiments. Controls without quercetin had the same concentration of DMSO as those with quercetin. The Selleckchem kinase library L1200 used for the HTS had been reformulated to be 1 mM stocks in DMSO.

GraphPad Prism v 7.04 was used for curve fitting and data analysis. Student’s *t* test was used for statistical analysis.

### Biochemical methods.

To analyze the binding between *Ct*PilB and MANT-ATP, MANT-ATP at 0.20 μM was mixed with *Ct*PilB at various concentrations in a 96-well plate in *Ct*PilB activity buffer (31). Samples were incubated at room temperature for 5 min before they were analyzed for fluorescence using an Infinite F200 PRO plate reader. The excitation wavelength (λ_ex_) was set to 355 nm and the emission wavelength (λ_em_) to 448 nm. For data analysis, the fluorescence of MANT-ATP and *Ct*PilB by themselves were subtracted for normalization. Their dissociation constant (*K_D_*) was calculated by fitting data to the Hill equation with the concentration of MANT-ATP and the fluorescence values as the variables. To analyze the competition with MANT-ATP for *Ct*PilB binding, ATP and ADP at different concentrations were mixed with *Ct*PilB at 0.50 μM and MANT-ATP at 0.20 μM in triplicates. The mixtures were incubated for 5 min at room temperature, and the fluorescence signals were measured and normalized by subtracting the fluorescence measured for *Ct*PilB and MANT-ATP. The IC_50_ of ATP and ADP on MANT-ATP binding were calculated using the 4-parameter logistic model (64) with the fluorescence and ATP or ADP concentrations as the variable. The inhibition constant (*K_i_*) was calculated using the Cheng-Prusoff equation (65).

To examine the effect of quercetin on the binding between MANT-ATP and *Ct*PilB, reactions with *Ct*PilB at 0.25 μM and MANT-ATP at 4 μM in triplicates were incubated at room temperature in the presence of quercetin at the indicated concentrations. Fluorescence was measured and normalized as described above. To analyze the effect of quercetin on the ATPase activity of *Ct*PilB, an endpoint ATPase assay was performed as previously described (31). The IC_50_ of quercetin on both MANT-ATP binding and ATPase activity were calculated as described above (64) with the concentrations of quercetin and either the fluorescence or the ATPase activity as variables.

### HTS based on fluorescence competition assay.

The HTS was conducted at the Virginia Tech Center for Drug Discovery (VTCDD) Screening Laboratory using the Agilent Bravo automated liquid-handling platform with 384-well assay plates (Greiner; 781101). Each assay well contained *Ct*PilB at 0.5 μM, MANT-ATP at 0.4 μM, and a library compound at 20 μM in 20-μl reaction mixtures in activity buffer. Fluorescence was measured using a SpectraMax M5 multimode microplate reader at room temperature. For the development and optimization of the HTS, ATP at 20 μM was used as the positive control because it was viewed as an inhibitor of the binding between MANT-ATP and *Ct*PilB, whereas samples without ATP were the negative controls for the absence of an inhibitor. The Z′ factor for this assay was calculated using the following equation (40): Z′ = 1 − [3 × (SD_+_ + SD_−_)/(μ_+_ − μ_−_)], where SD is the standard deviation and μ is the fluorescence intensity. The plus and minus subscripts refer to MANT-ATP and MANT-ATP with *Ct*PilB, respectively.

### Examination of the effects of quercetin on the bacterium M. xanthus.

Plate-based assays were used to examine motility and EPS levels. The starting materials for all experiments with M. xanthus were cells grown to log phase in CYE liquid media. M. xanthus cells were harvested and resuspended in MOPS buffer (10 mM morpholinepropanesulfonic acid [pH 7.6] and 2 mM MgSO_4_) to an optical density at 600 nm (OD_600_) of 5 (∼5 × 10^9^ cells/ml). For motility assays, 5 μl of the cell suspension was spotted onto either soft (0.4% agar) or hard (1.5% agar) CYE agar plates with the desired concentrations of quercetin. After incubation at 32°C for 4 days, the diameters of the colonies on these two sets of plates were examined to analyze T4P-depdendent and T4P-independent motility (45), respectively. In total, 12 colonies were measured per strain per set of conditions. To examine EPS levels, 5-μl aliquots of the cell suspension were placed onto CYE plates (1.5% agar) with calcofluor white (50 μg/ml) and EPS levels were assessed by fluorescence as previously described (51).

Liquid cultures were used to determine the MIC of quercetin and to examine the agglutination of M. xanthus cells. For the former, cells of YZ1674 were used to inoculate 5-ml CYE cultures with and without quercetin to a final OD_600_ of 0.05. These cultures were incubated in an orbital shaker-incubator at 32°C and 300 rpm for 24 h before their OD_600_s were measured. Cell agglutination was performed as described previously (26), with slight modifications. Briefly, cells were harvested, washed three times, and resuspended in agglutination buffer (MOPS buffer described above with 1 mM CaCl_2_). Cell suspensions were vortexed at the highest setting for 2 min to shear off their pili (53, 54). These bald cells were pelleted by centrifugation and resuspended in agglutination buffer to an OD_600_ of 0.9 with or without quercetin. Cell suspensions were transferred to cuvettes for the monitoring of OD_600_ over a time span of 3 h.

To assess the effect of quercetin on piliation, the pili on cells of M. xanthus strain YZ603 were sheared off as described above (53, 54). Bald cells were resuspended to an OD_600_ of 1 in ice-cold agglutination buffer with or without quercetin. After a 10-min pre-equilibrium period on ice, samples were incubated at 32°C for 20 min in the dark. Samples were then chilled on ice and centrifuged at 7,500 × *g* at 4°C for 10 min. These samples were vortexed for 2 min at the highest setting, followed by centrifugation at 16,000 × *g* for 4 min at 4°C. The pellets, which contained cells with their T4P sheared off, were resuspended to an OD_600_ equivalent of 5 in lysis buffer (50 mM Tris-HCl [pH 6.8], 2% [mass/vol] sodium dodecyl sulfate, and 1% [vol/vol] β-mercaptoethanol) as the pilin fraction. The supernatants, which contained the sheared-off T4P, were treated with MgCl_2_ at a final concentration of 100 mM for 1 h on ice. The T4P in the supernatant were precipitated by centrifugation at 21,000 × *g* for 15 min at 4°C. The resulting pellet was suspended to an OD_600_ equivalent of 10 with the lysis buffer as the T4P fraction. A lysate from whole cells (WC) with their T4P intact was also prepared in the lysis buffer with an OD_600_ equivalent of 5 for comparison. All above-described samples in the lysis buffer were boiled for 10 min and stored for future analysis.

For dot blotting using anti-pilin antibodies, a protocol was developed based on a previously described procedure (66). Briefly, the above-described WC and the pilin fractions were diluted 10-fold and the T4P fractions were diluted by a factor of 2. Four 1-μl aliquots of a sample were spotted onto a nitrocellulose membrane in a row and air dried. After blocking with blocking buffer (50 μM Tris base [pH 7.5], 150 μM NaCl, 5% [mass/vol] nonfat dry milk, and 0.1% [vol/vol] Tween 20), the membrane was incubated with rabbit anti-M. xanthus PilA serum (67) at a 1:10,000 dilution in TBST buffer (same as blocking buffer except with 0.1% [mass/vol] nonfat dry milk). This was followed by incubation with goat anti-rabbit antibodies conjugated to horseradish peroxidase (Thermo Fisher) at a 1:15,000 dilution in the same buffer. Blots were developed using SuperSignal West Pico PLUS chemiluminescent substrate (Thermo Fisher). The chemiluminescence signal was captured with a 1.5-s exposure using a ChemiDoc MP imaging system (Bio-Rad). For quantification, the pixel densities of the samples were analyzed using ImageJ (68).
