# Supplementary figures and images for: High-Throughput Screen for Inhibitors of the Type IV Pilus Assembly ATPase PilB
Source: mSphere. 2021 Mar 3;6(2):e00129-21. doi: 10.1128/mSphere.00129-21 (PMC8546689; doi:10.1128/mSphere.00129-21)

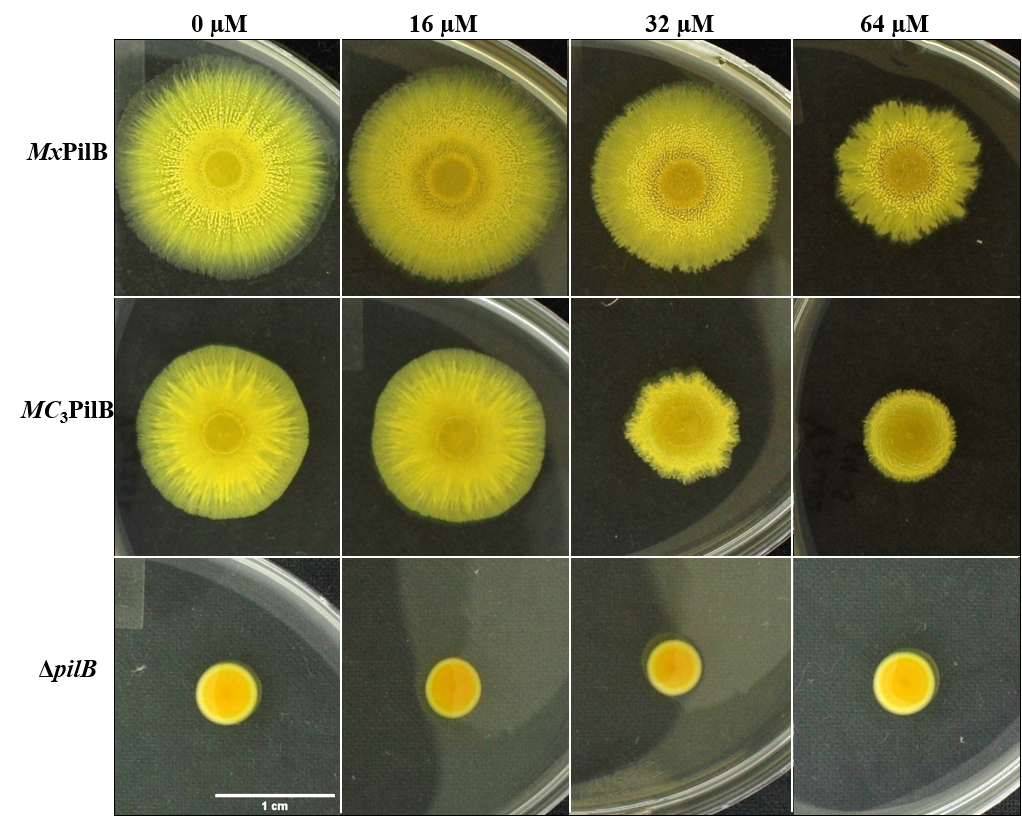

Supplement: FIG S1 [file msphere.00129-21-sf001.tif]

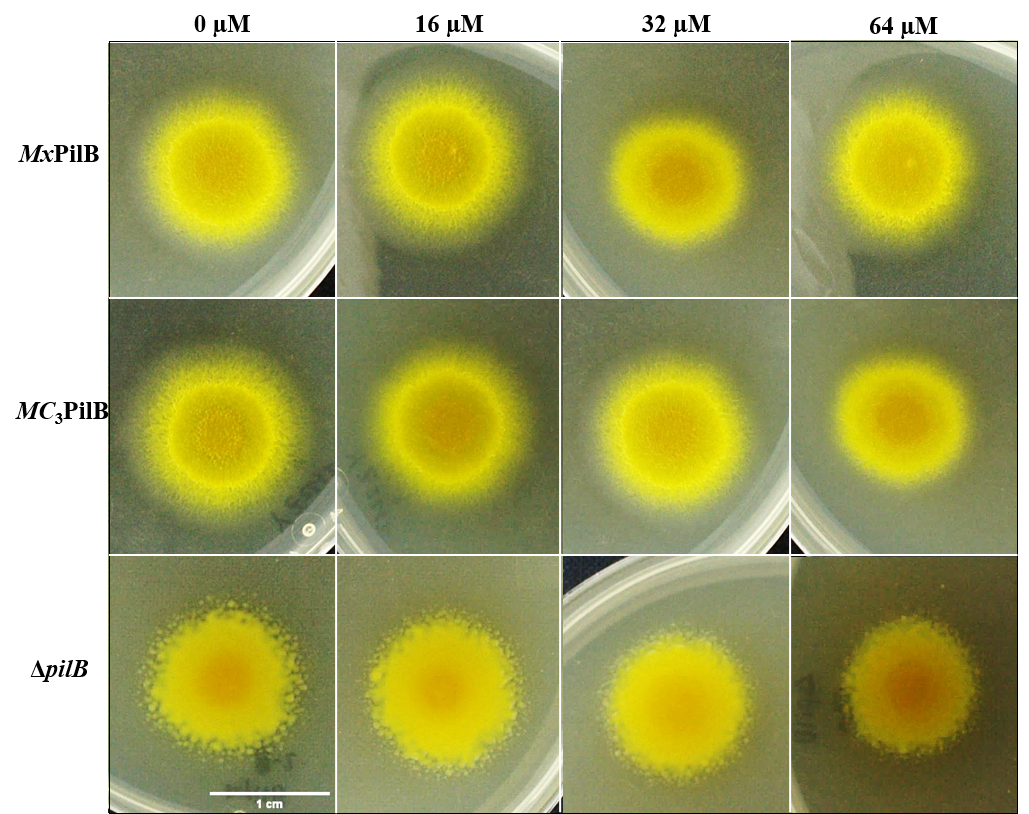

Supplement: FIG S2 [file msphere.00129-21-sf002.tif]

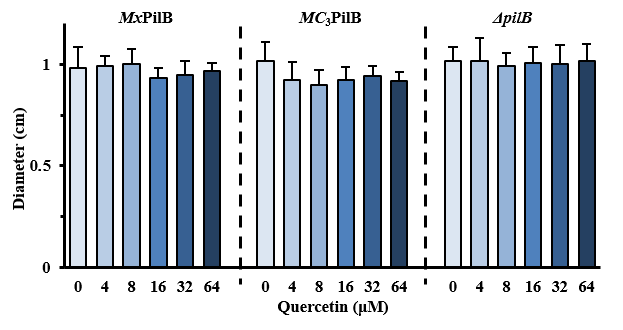

Supplement: FIG S3 [file msphere.00129-21-sf003.tif]

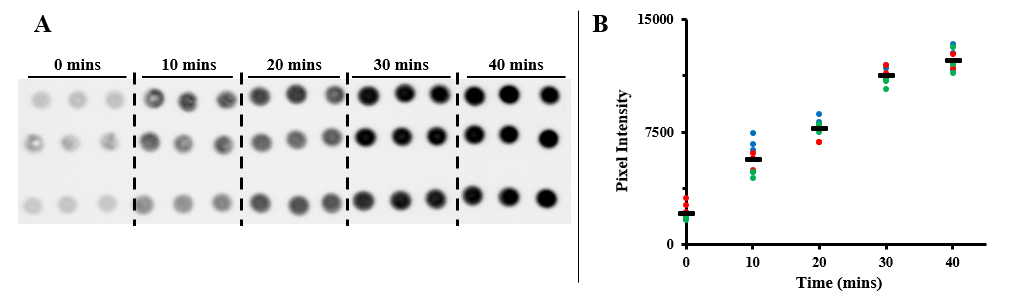

Supplement: FIG S4 [file msphere.00129-21-sf004.tif]

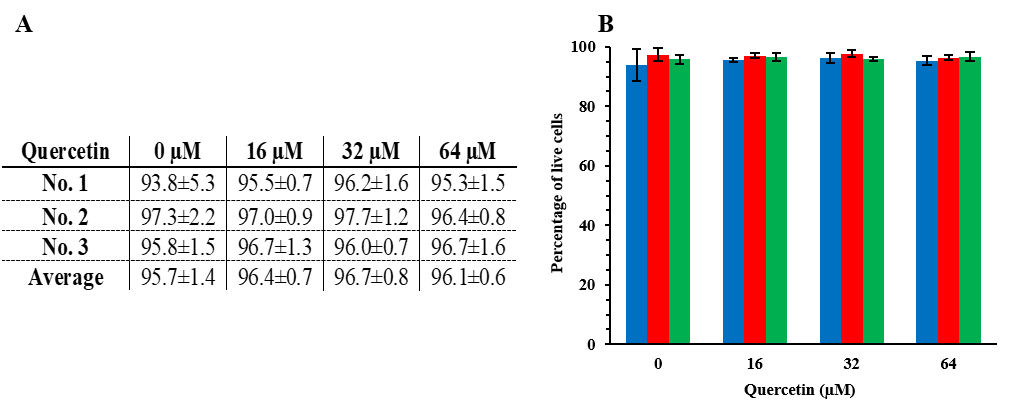

Supplement: FIG S5 [file msphere.00129-21-sf005.tif]
